# Differential Contributions of Vision, Touch and Muscle Proprioception to the Coding of Hand Movements

**DOI:** 10.1371/journal.pone.0062475

**Published:** 2013-04-23

**Authors:** Caroline Blanchard, Régine Roll, Jean-Pierre Roll, Anne Kavounoudias

**Affiliations:** 1 Aix-Marseille Université, CNRS, LNIA UMR 7260, Marseille, France; 2 Aix-Marseille Université, Marseille, France; University of Reading, United Kingdom

## Abstract

To further elucidate the mechanisms underlying multisensory integration, this study examines the controversial issue of whether congruent inputs from three different sensory sources can enhance the perception of hand movement. Illusory sensations of clockwise rotations of the right hand were induced by either separately or simultaneously stimulating visual, tactile and muscle proprioceptive channels at various intensity levels. For this purpose, mechanical vibrations were applied to the pollicis longus muscle group in the subjects’ wrists, and a textured disk was rotated under the palmar skin of the subjects’ right hands while a background visual scene was projected onto the rotating disk. The elicited kinaesthetic illusions were copied by the subjects in real time and the EMG activity in the adductor and abductor wrist muscles was recorded. The results show that the velocity of the perceived movements and the amplitude of the corresponding motor responses were modulated by the nature and intensity of the stimulation. Combining two sensory modalities resulted in faster movement illusions, except for the case of visuo-tactile co-stimulation. When a third sensory input was added to the bimodal combinations, the perceptual responses increased only when a muscle proprioceptive stimulation was added to a visuo-tactile combination. Otherwise, trisensory stimulation did not override bimodal conditions that already included a muscle proprioceptive stimulation. We confirmed that *vision* or *touch* alone can encode the kinematic parameters of hand movement, as is known for *muscle proprioception*. When these three sensory modalities are available, they contribute unequally to kinaesthesia. In addition to muscle proprioception, the complementary kinaesthetic content of visual or tactile inputs may optimize the velocity estimation of an on-going movement, whereas the redundant kinaesthetic content of the visual and tactile inputs may rather enhance the latency of the perception.

## Introduction

The Sherrington classification [1], which divides the human senses into the *proprioceptive*, *exteroceptive* and *interoceptive* categories, has been largely revisited and questioned. Currently, an extensive body of data has shown that the sensory modalities characterised as *exteroceptive* also contribute to the perception and control of human movement. For example, vision, which is classically described as an *exteroceptive* sense, has been found to play a *proprioceptive* role [2] especially based on the “vection” phenomenon studied during the 1970s [3], [4]. Movement of the whole visual field in front of stationary seated or standing subjects causes the subjects to feel that their body is leaning in the opposite direction, while the environment appears stationary. By using microneurographic [5]–[7] and psychophysical methods [8]–[11], several groups have provided neurophysiological and behavioural data supporting the idea that touch also plays a role in *proprioceptive* functions. In particular, it has been shown that cutaneous afferents from the skin that cover the dorsal part of the ankle [7], the knee [9], the fingers [8], [11], the dorsum [5], [6] and the palm of the hand [10] contribute to the detection and encoding of the kinematic parameters of imposed movements of these joints.

Under natural conditions, any limb movement gives rise to multiple sensory flows in which cutaneous, muscular and visual feedback play major roles. Questions thus arise as to how this multisensory feedback contributes to the encoding of movement parameters and how each sensory modality interacts with other. The combination of two sensory modalities, such as vision plus muscle proprioception [12]–[16], vestibular system plus muscle proprioception [17]–[19], vestibular system plus vision [20], [21], or touch plus muscle proprioception [8], [10], [11], [22], has generally been shown to improve the resulting perceptual or motor responses, whether it concerns the whole body or a single segment. The conclusions of these studies stress the need to integrate convergent inputs to properly assess body configuration and any changes that may occur.

However, this does not imply that these sensory sources contribute equally to these integrative mechanisms. In particular, van Beers *et al.* [14] have shown that subjects’ estimation of their hand location is based on a weighted combination of visual and muscle proprioceptive cues, according to the reliability of each sensory source. According to these authors, the central nervous system (CNS) relies more on muscle proprioception than on vision in depth direction. In contrast, vision is predominant in the azimuthal direction. Regarding cooperation between tactile and muscle afferents, Cordo et al. [11] also showed that both skin and muscle receptors contribute differently to the sense of movement, depending on the movement parameters that are being estimated. By creating a conflict between wrist muscle afferents and tactile inputs from the palm of the hand, we recently showed that their relative contributions to hand movement perception differ according to the velocity of the on-going illusion. Tactile information was found to override muscle proprioceptive information for estimating slow hand movements [10].

Even though under natural conditions, body movements involve simultaneous inputs from the tactile, muscle proprioceptive and visual systems, the manner in which the kinaesthetic inputs are co-processed across all three senses has not yet been investigated. Most studies of kinaesthesia have focused on bisensory integration. Therefore, one may wonder whether the addition of a third sensory source would impact the relative contribution of each of the three available sensory modalities and may improve kinaesthetic perception compared to the bimodal situation. One study by Jürgens & Becker [23] showed that in a visually structured environment, upright standing subjects turning about their vertical axis either passively through a rotating supporting platform or actively by means of small steps, more accurately identified their overall body angular displacement when the vestibular, visual and “*podokinaesthetic*” signals were generated simultaneously, rather than when only one or two of the cues were available. But this “*podokinaesthetic*” signal contents somatosensory information as well as efferent copy signal.

To determine the contribution of trisensory feedback to movement perception, we designed an experiment in which the visual, tactile and muscle proprioceptive systems were specifically stimulated, either alone or in combination. Vibratory stimulation applied to the subjects’ right wrist muscles and a rotating visual scene or a textured disk scrolling under the subjects’ right hands were used to create illusory movement sensations of hand rotation. In a first experiment, we showed that it was possible to induce similar kinaesthetic illusions by separately activating each of the three sensory systems and we established a relationship between stimulation intensity and the velocity of the resulting perceptions. In addition, since an involuntary motor response, the so-called antagonist vibratory response (AVR), is known to occur during vibration-induced illusions of an immobilised body segment [24]–[26], we investigated whether similar motor responses were associated with the visual- and tactile-induced illusory movements.

A second experiment was carried out to determine the extent to which kinaesthetic items of information from two or three different sensory sources are co-processed by the CNS to estimate the kinematic parameters of hand movements. To this aim the illusory movement sensations evoked by simultaneously applying two or three kinds of stimulation were compared with those evoked by the corresponding uni- or bisensory stimulation.

## Methods

Thirteen right-handed volunteers (8 women and 5 men; mean age: 30±8 years) with no history of neurological disease took part in the two experiments. All of the subjects gave informed consent, and the experimental procedures were carried out in accordance with the Helsinki Declaration. The local ethics committee (CCP Marseille Sud 1 #11/14) approved the study.

**Figure 1:**
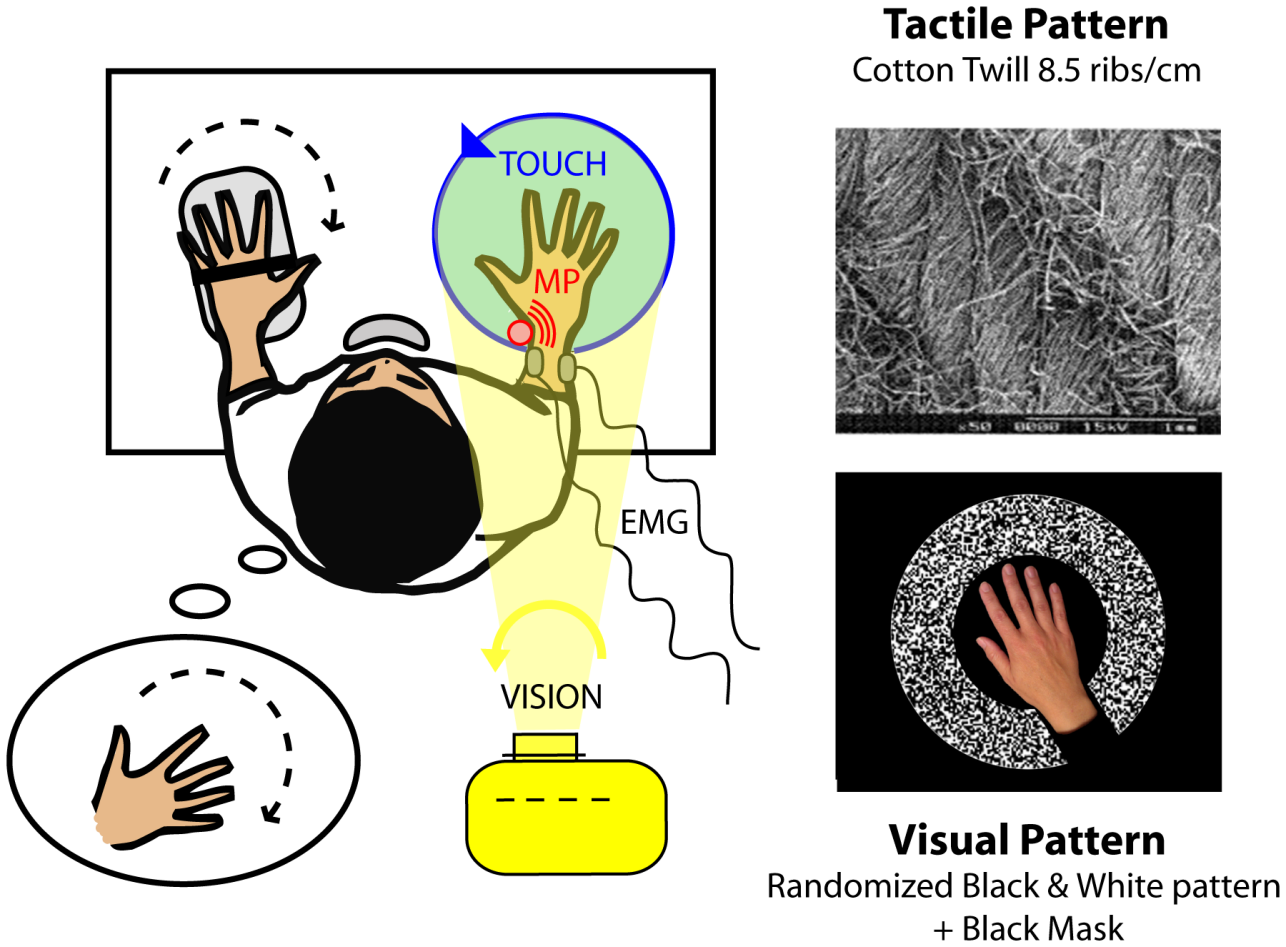
General experimental set-up. The subjects were exposed to a counterclockwise rotation of a textured tactile disk under their right hands (upper right) and/or a counterclockwise rotation of a visual scene projected via a video projector (bottom right) and/or a vibratory stimulation applied to the tendons of the pollicis longus muscle of their right wrists. The subjects held a potentiometer in their left hands to copy any illusory sensation perceived in their right hands. Potentiometric traces and EMG signals from the right extensor carpi ulnaris and pollicis longus muscles were recorded.

In both experiments, the subjects sat in an adjustable chair resting on the ground without wheels. They stood in front of a fixed table on which two adaptable arm-rests served to immobilise their arms and forearms. The subjects’ right hands rested on a disk (40 cm in diameter), and their left hands rested on a hand-rest equipped with a potentiometer. A chin-rest mounted on the table served as a support and immobilised the subjects’ heads (Fig. 1). All the mechanical constraints of the experimental set-up made the subjects’ right hands the only segments of the body free to move.

Throughout the experiments, shuttered glasses restricted the subjects’ visual field, allowing them to see only their own right hands in the centre of the field. A small abutment placed in the centre of the disk between the index and middle fingers prevented the hand from rotating with the disk. A mechanical vibrator was attached by elastic bands to the tendon of the pollicis longus muscle of the subjects’ right wrists (Fig. 1). Lastly, the subjects wore headphones to block outside noise. Although the experiments were carried out in darkness, the subjects were asked to close their eyes at the beginning of each trial, except for the conditions with visual stimulation, in which the subjects were asked to gaze at a target light in the centre of the disk.

In the present study, we aimed to induce a kinaesthetic clockwise hand illusion from the single and combined stimulation of three sensory sources: tactile, muscle proprioceptive and visual.

1. A tactile illusion was elicited using a disk that rotated under the subjects’ hands in a counterclockwise direction with a constant angular velocity of 5 to 40°/s. The disk was covered with cotton twill (8.5 ribs/cm, Fig. 1, top right), which is known to efficiently activate cutaneous receptors [27].
2. A muscle proprioceptive illusion was induced by 0.5-mm peak-to-peak mechanical vibrations applied to the pollicis longus tendon of the subjects’ right wrists in a frequency range from 20 to 80 Hz. The vibrator consisted of a biaxial DC motor with eccentric masses, forming a cylinder that was 5 cm long and 2 cm in diameter.
3. A visually induced illusory movement was obtained by means of a black and white pattern projected onto the disk from above the subjects. The pattern rotated around the centre of the disk with a constant counterclockwise angular velocity of 5 to 40°/s. To give the subjects the feeling that the pattern was moving in the background, i.e., under their hands, a black mask adjusted to the size of each subject’s hand was included in the video and prevented the pattern from being projected onto the subjects’ hands. A white fixation point was projected on the middle part of the subjects’ hands (Fig. 1, down right).

To assess the temporal and spatial characteristics of the illusory movements elicited by the various stimulation conditions, the participants were instructed to copy on-line any perceived right hand movements using the potentiometer in their left hand (Fig. 1). To facilitate the matching task, the axis of the potentiometer was collinear with the wrist axis. The subjects were asked to pay specific attention to the latency and velocity of the perceived movement. A beep informed the subjects when each trial was about to start or end. The potentiometric data were sampled at 1 kHz.

Surface electromyographic activity (EMG) was recorded from the extensor carpi ulnaris and pollicis longus muscles of the subjects’ right wrists (Delsys system - Bagnoli DE-2.1, Boston, MA, USA). The extensor carpi ulnaris and pollicis longus muscles are antagonist muscles involved in actual clockwise and counterclockwise hand rotation, respectively.

The electromyographic signals were amplified (x1000), band-pass filtered (20–500 Hz) and sampled at 1 kHz.

Any angular deviations arising from the potentiometer and the EMG activity from the pollicis longus and extensor carpi ulnaris muscles were recorded throughout each trial for each condition. Signal acquisition and stimulation delivery were performed using a National Instruments card (NI PCI-6229) and a specially designed software program implemented in Labview 8.2 environment. Regardless of the experimental condition, the stimulation started 750 ms after the onset of the data acquisition period and lasted for 9.25 s, until the end of the 10-s duration of the trial.

A training session was carried out to ensure that the subjects were able to use their left hands to accurately copy the illusory right hand movement that they experienced during the tactile, visual and/or muscular stimulation. This short period of familiarisation also served to train the subjects to divide their attention between the tasks of perceiving and copying the movements, which they had to perform simultaneously. The 13 subjects underwent two separate experimental tests on different days.

### Experiment 1

In experiment 1, we applied separate tactile, muscle proprioceptive and visual stimuli. The disk and the visual scene were rotated at 5, 10, 20, 30 and 40°/s, and the vibrator was used at 20, 30, 45, 60 and 80 Hz. These 5 stimulation levels are referred to as Xlow, Low, Medium, High and Xhigh intensities throughout the remainder of the paper. The subjects were tested under fifteen stimulation conditions throughout six sessions that were run on two different days to prevent the occurrence of long-lasting post-effects in subjects exposed to sustained sensory stimulation [28]. Each of the six sessions consisted of fifteen 10-second randomly delivered trials, with a 10-second rest interval between each trial.

### Experiment 2

In the second experiment, the tactile, muscle proprioceptive and visual stimuli were applied either separately or simultaneously at three intensity levels: Low, Medium and High. These intensity levels corresponded to rotation velocities of 10, 20 and 30°/s for the disk and the visual scene and to vibration frequencies of 30, 45 and 60 Hz for the vibrator. When 2 or 3 stimuli were delivered concomitantly, they were precisely synchronised and had the same duration.

The subjects were tested in three sessions on 3 different days. Each session consisted of 21 trial conditions lasting 10 seconds each, with a 10-second rest interval between the randomly delivered trials.

## Data and Statistical Analysis

### Potentiometric Data

Each potentiometer recording was first centred on the mean initial hand position calculated during the 750-ms phase prior to the stimulation onset. The direction, mean velocity and latency of the illusions copied by the thirteen subjects were then measured from the centred data. The velocity of the illusions (°/s) was calculated from the onset of the illusion up to the maximum angular deviation reached by the potentiometer using a linear regression method. The response latency (ms) was automatically determined at +2 standard deviations (SD) above the mean pre-stimulus level.

For each kind of unimodal stimulation (muscle proprioception, tactile, vision), one-way analyses of variance (ANOVAs) were used to test the effects of the stimulation intensity on the latency, velocity and gain (the ratio between the illusion velocity and the stimulation intensity) of the thirteen subjects’ illusory movement sensations. For each intensity level, pairwise comparisons using Student’s paired t-tests were used to compare the gains of the perceptual responses induced by the visual and tactile stimulation.

The velocities of the perceptual responses induced by multimodal (bi- and trimodal) and unimodal stimulation at the three levels of stimulation intensity were compared by means of two-way repeated-measures ANOVAs followed by post-hoc LSD Fisher tests.

In addition, the proportional enhancement or depression of the multisensory responses over the best unisensory response was computed using the “multisensory index” (MSI) as defined by Stein et *al.* [29]:

[(multisensory velocity –highest unisensory velocity )/highest unisensory velocity ]×100.

### EMG Data

Each electromyographic recording was first centred on the mean motor activity calculated during the 750-ms phase prior to the stimulation onset.

For each subject, we quantified the EMG responses in the extensor carpi ulnaris (ECU) and pollicis longus (PL) muscles by calculating the root mean squared (RMS) value of the centred EMG data averaged over the six trials. The response latency (ms) was automatically determined at +2 SD above the mean pre-stimulus level.

One-way repeated-measures ANOVAs were run to test the effects of stimulation intensity on the mean RMS values and the latencies of the thirteen subjects’ EMG signals.

All statistical analyses were performed using STATISTICA 8 software. The level of significance was set at 0.05.

## Results

### Experiment 1: Perceptual and Motor Effects of Single Muscle Proprioceptive Tactile and Visual Stimulation

**Figure 2:**
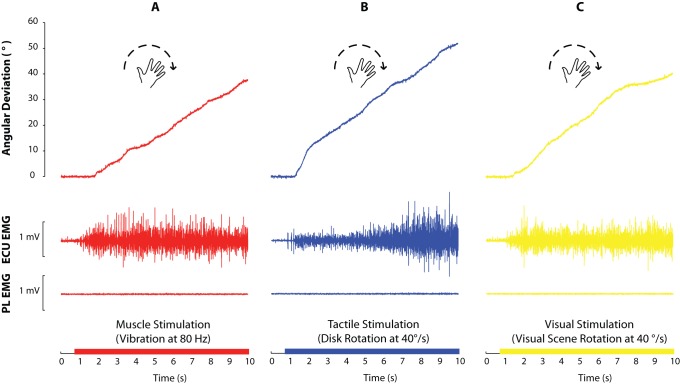
Typical recordings of clockwise angular deviations (°) copied by a participant and raw EMG activity (mV) in the extensor carpi ulnaris (ECU) and pollicis longus (PL) muscles during the three unimodal conditions. A- Muscle proprioceptive vibration at 80 Hz. B- Counterclockwise tactile disk rotation at 40°/s. C- Counterclockwise visual scene rotation at 40°/s.

The application of vibratory stimulation to the right pollicis longus muscle, rotation of the tactile disk in a counterclockwise direction or the projection of a counterclockwise rotating visual pattern under the subjects’ hands induced an illusory sensation of clockwise rotation of the right hand in all of the subjects. This kinaesthetic illusion was accompanied by a slight involuntary motor response in the extensor carpi ulnaris (ECU) muscle of the subjects’ right wrists. No changes occurred in the pollicis longus (PL) muscle activity (Fig. 2).

### Muscle Proprioceptive Stimulation (Fig. 3, left)

As expected, vibration applied to the pollicis longus muscle of the right wrist induced an illusory clockwise hand rotation in all of the subjects. An increase in the vibration frequency from 20 to 80 Hz resulted in a significant increase in the mean velocity of the resulting illusions (F(4,48) = 28.67, p<0.0001). Moreover, the mean illusion latency tended to decreased from 941 to 694 ms (F(4,48) = 2.38, p = 0.065).

The ratio between illusion velocity and vibration frequency was constant with respect to the vibration frequency (0.08 on average, F(4,48) = 2.33, p = 0.07), i.e., the illusion velocity increased linearly from 20 to 80 Hz.

**Figure 3:**
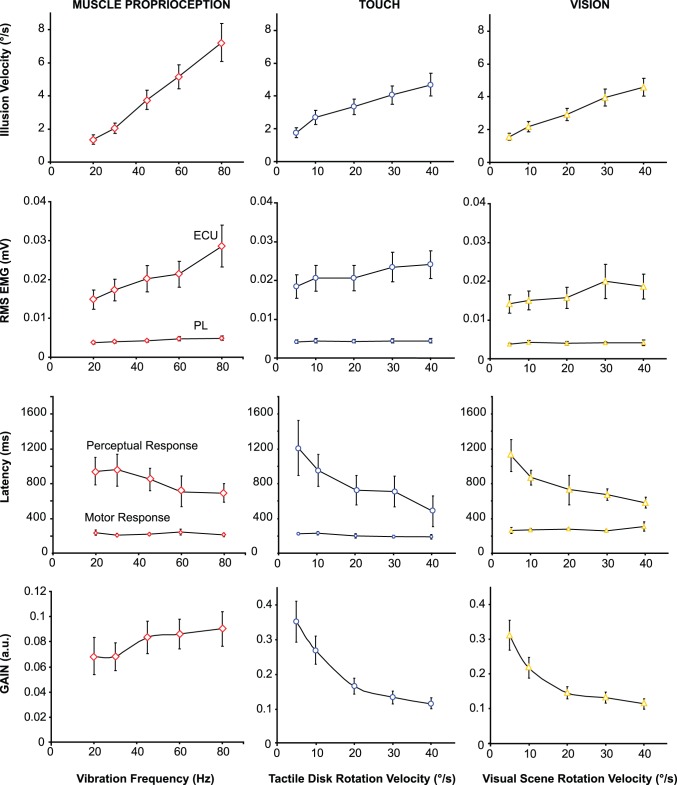
The effects of 5 levels of stimulation intensity on the perceptual and motor responses elicited by isolated proprioceptive (left column), tactile (central column) or visual (right column) stimulation. From top to bottom: Mean velocities (± S.D.) of the kinaesthetic illusions reproduced by the participants; Mean RMS (± S.D.) of EMG activity in the right extensor carpi ulnaris (ECU) and pollicis longus (PL) muscles;- Mean latencies (± S.D.) of the motor and perceptual responses; Mean gains (a.u.) of the perceptual responses (± S.D.), i.e., the ratio between the illusion velocity and the stimulation intensity.

The single-subject recordings displayed in Fig. 2 show that the vibration-induced illusions were accompanied by an early involuntary contraction of the extensor carpi ulnaris (ECU) muscle, whereas motor activity in the pollicis longus (PL) muscle remained unchanged. This motor response did not occur systematically at the lowest vibration frequency (20 Hz). For one of the 13 subjects, a motor response was not observed at any frequency level. For the other 12 subjects, the mean amplitude of the motor responses recorded in the ECU increased with vibration frequency (F(4,48) = 10.80, p<0.0001), but the latencies remained constant at approximately 230 ms on average (F(4,24) = 2.37, p = 0.08) (Fig. 3, left).

### Tactile Stimulation (Fig. 3, Central)

Counterclockwise scrolling of the tactile stimulus under the palmar skin of the subjects’ right hands induced a clockwise illusory hand rotation for all five velocities of tactile disk rotation tested (from 5 to 40°/s).

The mean velocity of the perceived hand rotation increased significantly with that of the disk (F(4,48) = 26.14, p<0.0001); at the highest velocities, a phenomenon of saturation occurred. We calculated the perceptual response in terms of the gain, i.e., the ratio between the illusion velocity and the actual velocity of the tactile disk. The gain was equal to one if the illusion and disk velocities were equal. As shown in figure 3, the higher the disk velocity, the lower the gain of the tactile response (F(4,48) = 22.62, p<0.0001), up to a plateau that was reached at the highest disk velocities (post-hoc 20°/s vs. 30°/s and 30°/s vs. 40°/s, p>0.05).

The latency of the tactile induced perceptual responses decreased significantly with disk velocity from a mean value of 1213 ms to 483 ms when the velocity of the disk rotated at 10°/s and 40°/s, respectively (F(4,48) = 8.22, p<0.0001).

The perceptual responses induced by the disk rotation were associated with an involuntary contraction of the extensor carpi ulnaris muscle, at an average of 220 ms (F(4,36) = 2.06, p = 0.11) after the beginning of the tactile stimulation, except in a few trials with the lowest stimulation intensity. The mean amplitude of the EMG activity increased with tactile disk velocity (F(4,48) = 7.08, p<0.0001).

### Visual Stimulation (Fig. 3, Right)

Counterclockwise rotation of the visual background under the subjects’ hands induced a clockwise illusory hand rotation at each of the five tested rotation velocities (from 5 to 40°/s). The velocity of the perceived hand movements increased significantly with that of the visual scene (F(4,48) = 38.66, p<0.0001).

Similarly to the tactile-induced illusory responses, the velocity of the visual-induced illusions did not vary linearly with the velocity of the projected pattern. The gain of the perceptual response decreased with the visual scene velocity (F(4,48) = 26.37, p<0.0001), and a phenomenon of saturation was observed for the highest velocities (post-hoc 20°/s vs. 30°/s and 30°/s vs. 40°/s p>0.05).

Pairwise comparisons showed that there were no significant differences between the gains of the visually and tactilely induced illusions for any of the applied stimulation velocities (Student’s paired *t*-tests: Xlow p = 0.50, Low p = 0.07; Med p = 0.22, High p = 0.74; Xhigh p = 0.84).

The mean latency of the visually induced illusions decreased significantly with visual scene velocity from 1125 to 584 ms when the velocity increased from 10 to 40°/s (F(4,48) = 10.14, p<0.0001).

The perceptual responses induced by rotation of the visual scene were also accompanied by an involuntary contraction of the extensor carpi ulnaris muscle, except for one subject who perceived the illusion without a motor response and for some trials conducted at the lowest stimulation velocities. This motor response occurred at an average of 300 ms (F(4,16) = 0.96, p = 0.46) after the beginning of the stimulation, with an amplitude that increased with the visual scene velocity (F(4,48) = 4.22, p = 0.005).

### Experiment 2: Kinaesthetic Effects of Combined Tactile, Visual and Muscle Proprioceptive Stimulation

According to the results of experiment 1, no significant differences were found between the mean velocities of the illusions elicited by either muscle proprioceptive, tactile or visual stimulation at any of the 5 tested intensity levels (F(4,48) = 1.73, p = 0.20). We therefore selected three of these five intensity levels to reduce the total number of sensory combinations to be tested. The lowest intensities, previously referred to as the X-low levels, were excluded since they did not always give rise to illusory perceptions in some subjects, and the highest intensities, the X-high levels, were removed to prevent any possible ceiling effect which would have prevented demonstrating a possible perceptual improvement associated with a tri-sensory stimulation compared to a bi-sensory one. Experiment 2 was therefore conducted using the Low, Medium and High intensity levels, corresponding to vibration frequencies of 30, 45 and 60 Hz for muscle proprioceptive stimulation and rotation velocities of 10°/s, 20°/s and 30°/s for the tactile and visual stimulation.

Using a two-way repeated-measures ANOVA, we verified that, as in the first experiment, similar illusory movements were evoked i.e. no significant differences were found between the mean velocities of the illusions induced by the 3 separate sensory kinds of stimulation (F(2,24) = 0.13, p = 0.88).

### Proprio-tactile Co-stimulation

**Figure 4:**
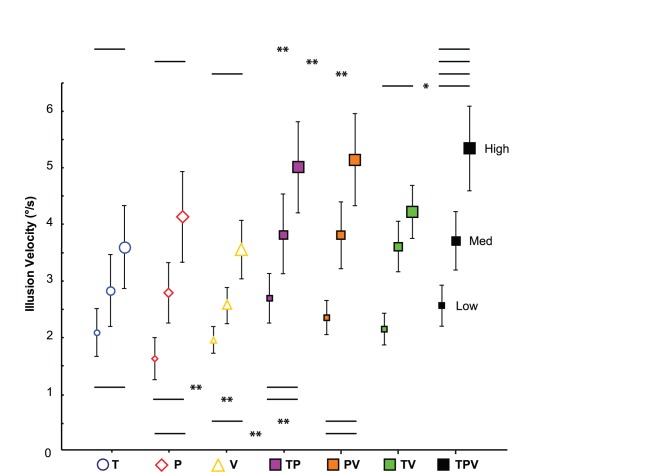
Comparison between the mean illusion velocities (± SEM) induced by unimodal, bimodal and trimodal stimulation applied at Low, Medium or High stimulation intensities. The unimodal stimulation conditions are represented by empty geometric figures: yellow triangle for tactile (T), red diamond for muscle proprioception (P) and blue circle for visual (V) conditions. The bimodal conditions are represented by solid geometric squares: purple squares for proprio-tactile (TP), orange squares for proprio-visual (PV) and green squares for visuo-tactile (TV) co-stimulation. The trimodal conditions are represented by solid black squares (TPV). Statistical data are p-values obtained by two-way ANOVAs (post-hoc tests,*p<0.05, **p<0.01; NS: Not significant).

**Figure 5:**
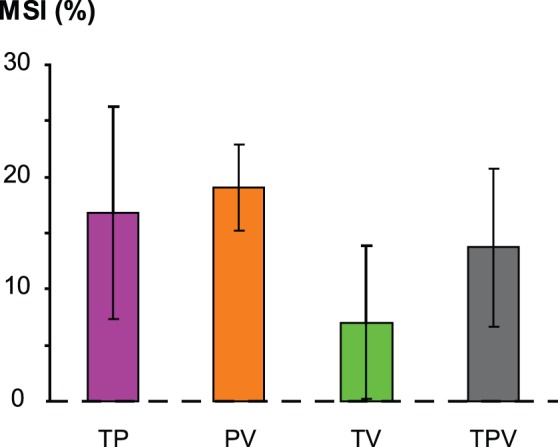
Proportional enhancement of the illusion velocities induced by bi- and trimodal stimulation over the fastest unisensory illusion. Histograms are the mean (± SEM) “multisensory index” MSI = [(multisensory velocity – highest unisensory velocity )/highest unisensory velocity ]×100.

The clockwise illusions observed during the bimodal proprio-tactile conditions were significantly faster than the illusions elicited by the tactile and muscle proprioceptive stimuli alone (type of stimulation: F(2,24) = 4.63, p = 0.02; post-hoc TP vs. P, p = 0.014, TP vs. T, p = 0.015; T vs. P, p = 0.97) (Fig. 4, *purple squares*). As calculated by the multisensory index (MSI), the velocities of the proprio-tactile responses were on average 16.8% higher than the velocities of the fastest unisensory responses observed for all the subjects (Fig. 5). Regardless of the stimulation type (uni- or bimodal), the direction of the resulting illusory hand rotations was always clockwise, and the velocity of the illusions increased with stimulation intensity (F(2,24) = 20.40, p = 0.000007).

### Proprio-visual Co-stimulation

When muscle proprioceptive stimulation was added to visual stimulation, the illusion velocity increased with the stimulation intensity, regardless of the intensity level (F(2,24) = 22.90, p<0.0001), and the perceived hand rotation was always faster than that elicited by unimodal conditions (type of stimulation: F(2,24) = 5.85, p = 0.009; post-hoc PV vs. P, p = 0.013; PV vs. V, p = 0.004; P vs. V, p = 0.63) (Fig. 4, *orange squares*). The velocity of the proprio-visual illusions was on average 19.1% than the most effective unisensory response (Fig. 5).

### Visuo-tactile Co-stimulation

When tactile stimulation was added to visual stimulation, the direction of the resulting illusory hand rotations was always clockwise and the illusion velocity increased with the stimulation intensity (F(2,24) = 26.14, p<0.0001) (Fig. 4, *green squares*).

Unlike the proprio-tactile and the visuo-proprioceptive stimulation, the mean velocities of the clockwise illusions induced by the visuo-tactile stimulation did not significantly differ from those elicited by the visual and tactile stimuli alone (F(2,24) = 2.20, p = 0.13). As shown in figure 5, the MSI index for the visuo-tactile conditions was very low, only 7% on average. In contrast, the visuo-tactile illusions occurred earlier than those induced by unisensory stimulation (type of stimulation: F(2,22) = 10.76, p = 0.0005; post-hoc TV vs. T, p = 0.002, TV vs. V, p = 0.0002, T vs. V, p = 0.37) (Table 1).

**Table 1:** Mean Latencies (ms) ±S.D. of kinaesthetic illusions induced by uni-, bi- and tri-modal stimulation applied at Low, Medium or High intensity level.

	STIMULATION						
	*T*	*P*	*V*	*TP*	*TV*	*PV*	*TPV*
*Low*	905±186	1075±199	1010±125	630±84	689±83	921±108	789±117
*Medium*	739±140	1095±175	829±108	832±134	539±71	749±98	563±77
*High*	697±109	985±186	685±46	647±104	405±39	703±101	464±70

### Visuo-proprio-tactile Co-stimulation

When the tactile, visual and muscle proprioceptive modalities were stimulated simultaneously, the velocity of the perceived movement was always higher than the illusion velocities elicited by any of the three corresponding types of single stimulation (type of stimulation: F(3,36) = 4.65, p = 0.008; post-hoc TPV vs. T, p = 0.008; TPV vs. P, p = 0.007; TPV vs. V, p = 0.002) (Fig. 4, *black squares*). For the 3 intensity levels, the velocity of the trimodal responses was about 13.7% over the fastest unisensory responses (Fig. 5).

The latencies of the perceptual responses decreased significantly for the trimodal conditions compared to those of the unimodal conditions, except for the tactile stimulus (type of stimulation: F(3,33) = 4.73, p = 0.007; post-hoc TPV vs. T, p = 0.16, TPV vs. P, p = 0.0008; TPV vs. V, p = 0.038) (Table 1).

In addition, the responses induced by trisensory stimulation were significantly faster than those elicited by bisensory stimulation for the visuo-tactile combination (F(1,12) = 6.88, p = 0.02) at the highest stimulation intensity (TPV high vs. TV high, p = 0.0004; TPV med vs. TV med, p = 0.76; TPV low vs. TV low, p = 0.13). In contrast, for the 3 intensity levels, no significant differences were found between the illusion velocities induced by the trimodal and the proprio-tactile (p = 0.91) or proprio-visual (p = 0.52) stimulation conditions (Fig. 4).

## Discussion

### Activation of the Perceptual-to-motor Loop by Sensory Feedback from Different Origins

The finding that a similar illusory movement was evoked by stimulating any one of the three sensory modalities tested supports the general assumption that vision and touch also convey kinaesthetic messages, as is known to occur for muscle proprioception. However less is known about the possible motor effects associated with such perceptual responses.

Regarding muscle proprioception, the motor responses referred to as “antagonist vibratory responses” (AVRs) in the study of Roll *et al.* [24] are known to occur together with a vibration–induced illusion of movement. Tonic activity arises in the muscle group antagonistic to that vibrated, i.e., the muscle that would have been contracted if the movement was actually performed [25], [26]. We consistently found that a clockwise illusory hand rotation induced by vibration of the pollicis longus muscle was accompanied by involuntary motor activity in the extensor carpi ulnaris muscle, the muscle that is typically involved in a clockwise hand movement. This confirms that the perception of movement generates appropriate corresponding motor activity.

Interestingly, at an average of 250 ms after the onset of stimulation, we also observed EMG activity in the same extensor carpi ulnaris muscle group during clockwise illusory hand rotations induced by either tactile or visual stimulation, i.e., visual inputs, as well as skin and muscle afferents from the hand gave rise to equivalent perceptive and associated motor effects. Moreover, these effects increased with stimulation intensity, as previously demonstrated by Calvin-Figuiere *et al.* [26] for the muscle proprioceptive system.

These findings led us to conclude that the motor responses associated with an illusory movement result from a perceptual-to-motor transformation at a high level, rather than from spinal reflex mechanisms.

These results are also in agreement with those of Romaiguere *et al.* [30], which showed that transcranial magnetic stimulation of the sensorimotor cortex during vibratory stimulation of the wrist muscle groups affects both illusory movement perception and the motor response. Using magnetoencephalography, Casini *et al.* [31] demonstrated that the primary motor cortex was activated as early as within the first 200 ms following wrist muscle vibration and that this activation occurred only when the muscle proprioceptive stimulation induced a hand movement illusion. Other fMRI studies have shown a concomitant activation of sensory- and motor-related brain regions during the perception of a vibration-induced illusory movement, including activation of the contralateral primary somatosensory and motor cortices, the contralateral premotor cortex, the bilateral cingulate and supplementary motor areas, the bilateral posterior parietal cortex and the ipsilateral cerebellum [28], [32], [33]. We also recently found large areas of overlap in sensorimotor activation maps when subjects experienced an illusory hand rotation evoked by rotating a disk under the subjects’ hands or by vibrating a wrist muscle group. This indicates that whether they are induced by stimulation of the skin or the muscle, kinaesthetic illusions share a common associated sensorimotor cerebral network [34].

Interestingly, during a passively imposed movement, it has been demonstrated that an involuntary contraction occurs in the shortened muscle [35], [36]. This so-called “shortening reaction” first reported by Westphal [37] is known to be strongly affected following cortico-spinal damage. It has been proposed that this “shortening reaction” might be generated by the cortical drive to inhibit the antagonist short latency reflex and to therefore assist the movement passively imposed [35] as well as the motor effects associated with visual-, tactile- and vibration-induced illusory movements.

Whether it concerns the central or peripheral level, these data provide compelling evidence for a powerful link between kinaesthesia and action, stressing that perception of a passive or illusory movement cannot emerge from pure sensory-based processing without a motor-related component, regardless of the sensory source by which the motion is perceived.

### Velocity-dependent Integration of Visual, Muscle Proprioceptive and Tactile Feedback for Hand Motion Perception

As expected, the perceptual responses to all three types of sensory stimulation varied according to the stimulus intensity. The responses occurred earlier and at higher velocities as the stimulation intensity increased.

It is worth noting that we observed almost identical velocity profiles for the perceived movement induced by the tactile and visual stimuli. In both cases, the gains of the perceptual responses were clearly below unity. This finding likely results from the motion information provided by visual or tactile stimulation being in conflict with the unchanged hand position information provided by the muscle proprioceptive channel. In addition, for both visual and tactile stimulation, the gain of the resulting illusory movements decreased as the stimulation velocities increased. Decreases in the gain of visually induced illusory movements have previously been reported for postural sensations evoked in standing subjects. It has been well documented that subjects standing upright in front of a visual scene rotating at a constant velocity feel that their whole bodies are rotating in the opposite direction with a velocity increasing with that of the visual motion up to 30°/s. Above this saturation value, the velocity of visually induced postural illusions decrease [4], [38]–[43]. We had already consistently observed this decrease in the gain of illusory hand movements induced by the tactile stimulation [10]. The finding that one experiences similar hand movement perceptions regardless of whether it is based on either visual or tactile sensory inputs argues for a redundant kinematic content of theses two sensory signals. In addition, the sensory message of movement provided by either visual or tactile cues can be interpreted as a movement of the body, the environment or both, whereas muscle proprioception relates unambiguously to the configuration of one’s own body and any changes that may occur in its position. Given that the perception of fast movements is more likely attributable to environmental displacement than to self-body movements, the saturation phenomenon that we observed for the fastest tactile and visual types of stimulation may be interpreted as a central re-weighting of these ambiguous messages. Dokka *et al.’s* [44] results support this hypothesis by showing that during visually induced postural illusions, the CNS integrates visual and non-visual inputs by assigning a higher probability to slow, rather than fast, movements.

Another possible explanation could be that the decrease in gain is due to intrinsic limits of the visual and tactile sensory systems, with a lower capability for encoding fast moving stimuli in comparison to slow moving stimuli. However, this interpretation is not consistent with the results of psychophysical studies analysing the velocity discrimination of visual [45], [46] or cutaneous [47], [48] stimuli. Moreover, we know from microneurographic recordings the responses of cutaneous mechanoreceptors to superficial brushing of the skin by the same textured stimulus that was used in the present study [27]. No saturation was observed in the frequency of discharges of the cutaneous receptors, up to a brushing velocity of 150°/s”.

In contrast, no saturation was observed in response to muscle stimulation in the frequency range used in the present experiment; the perceived velocity of the kinaesthetic illusions increased linearly from 20 to 80 Hz. This is consistent with studies showing that the application of low-amplitude (0.2–0.5 mm) mechanical vibration to a muscle tendon mainly activates the muscle spindle primary endings (Ia), with a discharge frequency that increases linearly with vibration frequency up to 80–100 Hz [49]–[51]. By imposing passive rotation of the metacarpophalangeal joint, Grill & Hallett [5] consistently found a linear increase in the discharge frequency of Ia afferents when the velocity of the passive movement progressively increased from 5 to 80°/s.

To conclude, our findings suggest that as the stimulus velocity increases, the weights of visual and tactile information for estimating the kinaesthetic parameters of hand movements decrease to favour less ambiguous sensory sources, such as muscle proprioception in the motor apparatus.

### Muscle Proprioception as the Key Vector of Kinaesthetic Information

As equivalent illusions of hand movement can be elicited by separate visual, tactile and muscle proprioceptive sensory channels, one might expect that these three sensory inputs could be used indiscriminately by the CNS for determining the kinematic parameters of any on-going hand movement. However, under multimodal stimulation conditions in which two or three sensory sources are available, illusory perception analyses show that the CNS does not rely equally on the different types of sensory information.

For example, the addition of muscle proprioceptive stimulation to a visual or tactile stimulus enhanced the subjects’ perception of the movement velocity of their hands, whereas no enhancement was observed for visuo-tactile co-stimulation. The subjects always reported that their hand movements were slower than the real rotation of the visual and tactile stimulation, but their illusions were stronger and faster when proprio-tactile and proprio-visual stimulation were used, compared to those elicited by each type of stimulation alone.

To explain the discrepancy between the bimodal conditions, one can hypothesise that integrative processing varies according to the nature of the sensory sources at work, depending on the behavioural context. Proprioceptive information from the wrist muscles can remove the ambiguity regarding whether visual or tactile motion originates from the self or from the environment. Thus, tactile and visual information may be co-processed with muscle proprioceptive signals by the CNS, in order to optimize the perception of hand movements. When only visual and tactile feedback is available, the CNS might favour the most behaviourally relevant type of information and use the remaining information to confirm its previous interpretation. However, a detailed analysis of the time course of the perceptual responses showed that the latencies of the illusions induced by the visuo-tactile stimulation were always lower to those of the two corresponding unimodal conditions. Moreover, the subjects generally reported that the illusory sensations, including those induced by visuo-tactile co-stimulation, were more salient under the bimodal conditions compared to the unimodal conditions. These findings seem to rule out the possibility that estimations of hand movements may be based on only one of these two sensory inputs.

We consistently found that trimodal stimulation resulted in faster illusory perceptions in comparison to those induced by the visuo-tactile combination at the highest intensity level, but the perceptions were not faster than those provoked by the proprio-visual and proprio-tactile combinations at any intensity level. In other words, the addition of a third sensory stimulus to a bimodal stimulus improved the resulting movement perception only when a strong muscle proprioceptive signal was added to a bimodal visuo-tactile condition.

As the visual and tactile types of stimulation were found to induce similar perceptual responses, one might expect that the CNS could extract identical kinematic information from these two sensory sources, which would fail to improve the velocity of the perceived movement. In contrast, the CNS might take advantage of the kinaesthetic content of a muscle proprioceptive input to disambiguate the origin of an on-going movement (self vs. environment) and to optimize its velocity estimation. Indeed, the muscle proprioceptive motion signal can be considered in the situation tested here as providing 'absolute' hand motion information, because the body is known by the subjects to be stationary in space and the hand is linked to this frame of reference via the arm. This does not apply to the touch cues and the visual motion cues, which provide relative motion information.

Whereas the occurrence of visual and tactile information are context-dependant, the insertion of the proprioceptive system into the motor apparatus itself and its high sensitivity to any changes in muscle length make this sensory system likely the more reliable one to encode kinematic parameters of body movement. However, after a transient or a definitive loss of proprioceptive afferents, the weight of the remaining intact sensory channels improves to partly compensate for sensorimotor deficits. For instance, visual-dependency is known to increase dramatically in proprioceptively deafferented patients caused by sensory neuropathy [52], [53].

To conclude, visual, tactile and muscle proprioceptive inputs contribute jointly but unequally to the human perception of movement depending on the velocity of the on-going movement and the nature of the sensory information available. The combination of complementary kinds of information can improve the perception of hand movement velocity, whereas the combination of redundant types of information has the potential to enhance the quality of the perception without improving its velocity estimation. Also, that consistent motor responses have been evidenced during perception of illusory movements regardless of the sensory source by which they were induced strengthens the general assumption of a close linkage between perception and action at a high level of the CNS. Further studies should be conducted to determine the extent to which these three kinds of sensory stimulation can activate a common sensorimotor network to result in similar perceptive and motor effects as observed in this study.
